# Associations of LH and FSH with reproductive hormones depending on each stage of the menopausal transition

**DOI:** 10.1186/s12905-023-02438-5

**Published:** 2023-05-25

**Authors:** Takako Kawakita, Toshiyuki Yasui, Kanako Yoshida, Sumika Matsui, Takeshi Iwasa

**Affiliations:** 1 Department of Obstetrics and Gynecology, Institute of Biomedical Sciences, The University of Tokushima Graduate School, Tokushima, Japan; 2 Department of Reproductive and Menopausal Medicine, Institute of Biomedical Sciences, The University of Tokushima Graduate School, Tokushima, Japan; 3 Department of Obstetrics and Gynecology, Tokushima Red Cross Hospital, Tokushima, Japan

**Keywords:** LH, FSH, Menopausal transition, Androgen

## Abstract

**Introduction:**

Associations of luteinizing hormone (LH) with androgens during the menopausal transition and associations between follicle-stimulating hormone (FSH) levels and various diseases related to reproductive hormones in postmenopause have received much attention. LH and FSH are also known to be associated with activities of enzymes related to reproductive hormones. We examined the associations of LH and FSH with androgens and estrogens in each stage of the menopausal transition according to a classification from menopausal transition to postmenopause.

**Methods:**

This study was a cross-sectional design. We basically used the Stage of Reproductive Aging Workshop (STRAW) + 10. We divided the 173 subjects into 6 groups according to menstrual regularity and follicle-stimulating hormone level: mid reproductive stage (Group A), late reproductive stage (Group B), early menopausal transition (Group C), late menopausal transition (Group D), very early postmenopause (Group E) and early postmenopause (Group F). Levels of LH, FSH, dehydroepiandrosterone sulfate (DHEAS), estradiol, estrone, testosterone (T), free T, androstenedione and androstenediol were measured.

**Results:**

In Group A, LH showed significant positive correlations with androstenedione and estrone. In Group D, LH was positively associated with T and free T and was negatively associated with estradiol. In Groups B, C, D and F, LH showed significant positive correlations with FSH, and there was a tendency for an association between LH and FSH in Group E. FSH was associated with estradiol but not with estrone in Groups C and D.

**Conclusion:**

The associations of LH and FSH with reproductive hormones are different depending on the stage of the menopausal transition.

**Trial registration:**

Trial registration number 2356-1; Date of registration: 18/02/2018, retrospectively registered.

## Introduction

**Fig. 1:**
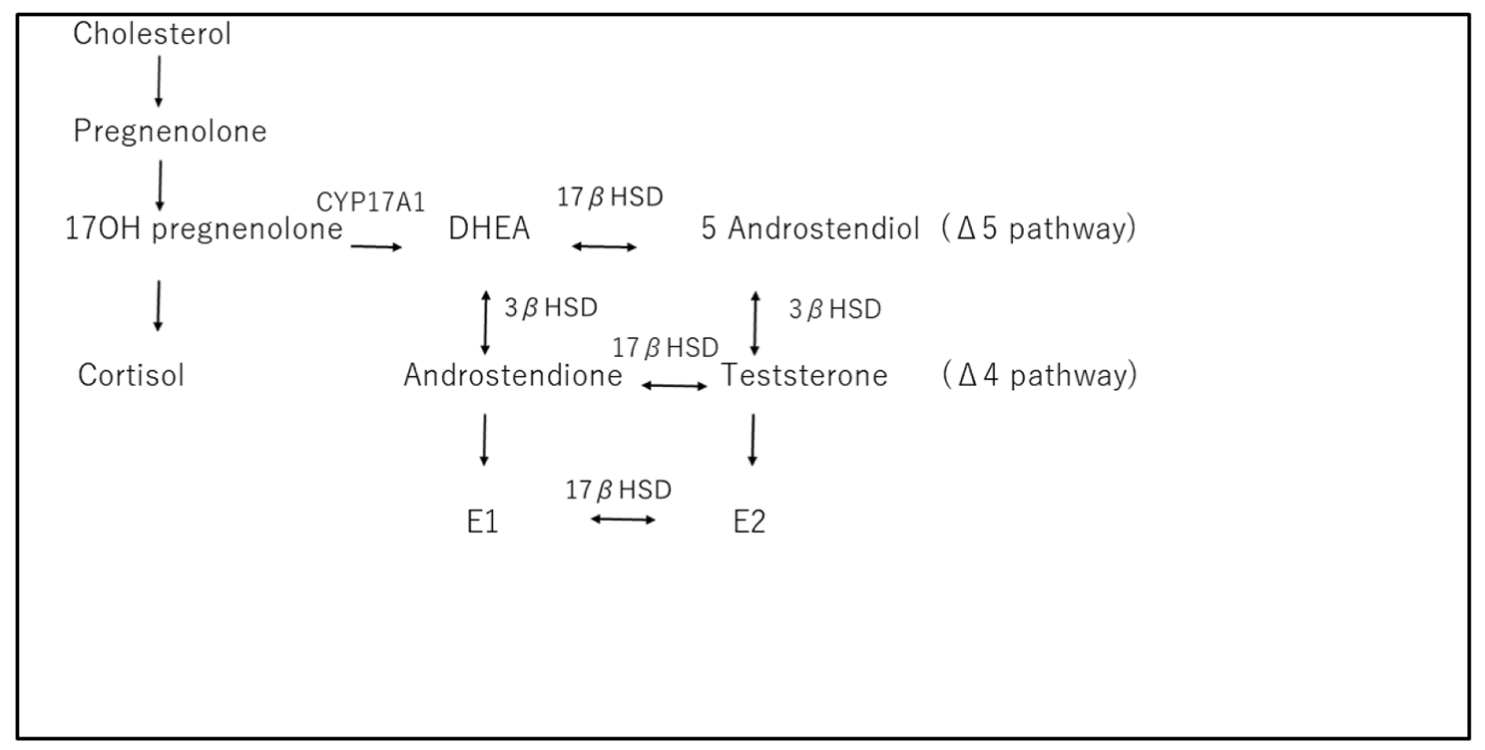
Delta-4 and delta-5 pathways

During the menopausal transition, the production of luteinizing hormone (LH) and follicle-stimulating hormone (FSH) in the pituitary increases to compensate for the declining estradiol levels due to a decrease in ovarian function. Based on changes in FSH trajectory accelerations and decelerations and rates of change, four menopausal transition stages bounding the final menstrual period and eight epochs in chronological aging from ages of 28 to 60 years have been defined [1]. Also, in women aged 42–52 years, three FSH trajectories over the menopausal transition have been identified [2]. Previous studies have shown that FSH receptors are distributed in various tissues including the bone [3, 4], liver [5] and vessels [6] as well as the ovary. It has been shown that FSH has extragonadal actions and that FSH levels are associated with various diseases and with metabolism in postmenopause [7, 8]. On the other hand, LH receptors are also distributed in not only the ovary but also the adrenal gland [9], brain [10], skin [11] and bladder [12]. It has been reported that an LH level of less than 41 U/L showed a positive correlation with dehydroepiandrosterone sulfate (DHEAS) level in postmenopausal women but not in women during the menopausal transition [13], suggesting that DHEAS production from the adrenal gland may be stimulated by highly elevated LH levels [13]. Although much attention has been focused on the delta-4 steroidogenic pathway that produces cortisol, androstendione and testosterone, longitudinal studies have suggested that the delta-5 steroidogenic pathway that produces DHEA as shown in Fig. 1, DHEAS and androstenediol may play a more important role in women’s healthy aging [14, 15]. It has been reported that gonadotropins such as LH and FSH change the activities of enzymes, including cytochrome P450 (CYP) 17 A, 3β-hydroxysteroid dehydrogenase (HSD) and 17β-HSD, that act in the delta-4 and delta-5 steroidogenic pathways [16–19]. During the menopausal transition, increases in LH and FSH levels change the activities of enzymes and might be associated with changes in the levels of reproductive hormones.

17OH pregnenolone: 17-hydroxyprogesterone, CYP17A1: cytochrome P450 17A1, 17βHSD: 17β-hydroxysteroid dehydrogenases, 3βHSD: 3β-hydroxysteroid dehydrogenase.

In 2012, the Stage of Reproductive Aging Workshop (STRAW) + 10 staging system was revised. The revised staging system improves the comparability of studies of midlife women and is widely considered as the gold standard for characterizing reproductive aging through menopause [20]. Based on the STRAW staging system, we found associations of androstenediol levels related to the delta-5 steroidogenic pathway with estrogen and androgen in women during the menopausal transition in a previous study by using liquid chromatography mass spectrometry (LC-MS/MS) with high sensitivity and high specificity [21]. Associations of LH with androgens during the menopausal transition have been shown, but there has been no report on the associations between FSH and androgens including androstenediol. The associations of LH and FSH with reproductive hormones related to the adrenal gland may differ according to each stage in the STRAW classification. To our knowledge, information on the associations of gonadotropins, including LH and FSH, with androgens and estrogens in premenopausal women, women during the menopausal transition and postmenopausal women is scarce. Thus, we aim to elucidate the associations of LH and FSH levels with reproductive hormone levels in premenopausal women, women in the menopausal transition and postmenopausal women.

## Subjects and methods

### Subjects

We recruited 173 Japanese women for this cross-sectional study from the outpatient clinic of the Department of Obstetrics and Gynecology in Tokushima University Hospital. These women visited a specialized health care outpatient clinic for consultation and for management and treatment of various conditions including decrease in bone mineral density, dyslipidemia and menopausal symptoms. Women who had received hormone therapy in the past year were excluded. At the time of the visit to the outpatient clinic, we obtained information on menstrual frequency and flow or years since the final menstrual period (FMP) and we measured FSH level and determined the stage of menopausal transition. Based on the STRAW staging system [20], we divided the subjects into 6 stages by menstrual regularity and FSH level: (1) women with a regular menstrual cycle (25–35 days per cycle) and normal FSH level (mid reproductive stage, Group A, n = 21), (2) women with a regular menstrual cycle and elevated FSH level (> 10 mIU/ml) (late reproductive stage, Group B, n = 22), (3) women with an irregular menstrual cycle and elevated FSH level (> 10 mIU/ml) (early menopausal transition, Group C, n = 23), (4) women who had an irregular menstrual cycle in which the interval of amenorrhea was more than 2 months and who had elevated FSH level (late menopausal transition, Group D, n = 35), (5) women for whom less than 1 year had passed since menopause (very early postmenopause, Group E, n = 30), and (6) women for whom more than 1 year and less than 5 years had passed since menopause (early postmenopause, Group F, n = 42). Informed consent for participation in this study was obtained from each woman. The Ethics Committee of Tokushima University Hospital approved the study.

### Measurements

Blood in women with menstruation was drawn from 9:00 to 12:00 during a period of 3–7 days after the commencement of menstruation. Blood samples for measurements were obtained by venipuncture and drawn into tubes. They were frozen at -40℃ until used for analysis. Levels of LH, FSH, estradiol and testosterone (T) were measured by a chemiluminescent immunoassay. DHEAS level was measured by a chemiluminescent enzyme immunoassay. Levels of androstenedione and free T were measured by a radioimmunoassay. The intra- and inter-assay coefficients (CVs) for DHEAS, T, free T, estradiol, androstenedione, LH and FSH were less than 10%. Serum androstenediol and estrone concentrations were measured by using LC-MS/MS, and the measurements were described in our previous report (21). The intra- and inter-assay CVs for androstenedione measurements were 2.3–2.9% and 4.4–6.3%, respectively. The intra- and inter-assay CVs for estrone measurements were 2.0-2.3% and 3.0-3.2%, respectively. The sensitivity of the assay was 0.01 ng/ml for androstenediol and estrone.

### Statistical analysis

All statistical analyses were performed by using SPSS statics version 20.0 (IBM, Armonk, New York). Data are presented as medians with 25th and 75th ranges and LH/FSH ratio is shown as a mean. The Kruskal-Wallis rank test was used to compare differences between different menopausal stages, and Bonferroni adjustment was used for a multiple comparison test. Correlations between variables were assessed by Spearman’s rank correlation analysis.

## Results

Background characteristics of the subjects are shown in Table 1. Median body mass index (BMI) ranged from 20.1 to 22.5 kg/m^2^, and there was no significant difference in BMI among the groups. BMI in one woman was more than 30.0 kg/m^2^ (Table 1). None of the study subjects had dyslipidaemia or diabetes, or hypertension requiring any medication.

**Table 1:** Clinical characteristics of the subjects

		Group						P value
		A	B	C	D	E	F	
Number		21	22	23	35	30	42	
Age	years	43.5 41.50–46.50	47 44.50–49.00	47* 46.00–49.00	49* 46.50–50.50	51* 49.50–53.00	53* 51.75-54.00	< 0.001
Height	cm	155.0 152.25–157.00	158.0 154.00-160.85	160.4 158.93–163.00	158.7 156.63–160.00	158.0 154.00-160.00	156.5 155.00-159.05	0.189
Weight	kg	50.1 47.70-56.25	55.9 50.5-57.65	56.0 52.65–58.53	59.0 52.00-61.20	52.0 49.40–57.40	53.1 50.00-57.15	0.182
BMI	kg/cm²	20.1 19.50–21.50	21.4 20.00-23.55	21.8 20.51–23.13	22.5 20.31-25.00	21.9 20.09–22.79	21.7 20.30-23.53	0.379
LH	mIU/ml	3.7 2.83- 5.90	7.2 4.05–13.28	8.8 7.7- 26.3	30.05* 17.48- 35.78	33.30* 20.80- 43.90	30.55* 23.5- 38.48	< 0.001
FSH	mIU/ml	5.6 4.80- 8.00	14.05 11.15- 35.18	23.9 13.5- 42.9	56.95* 30.55- 87.35	93.40* 70.70- 110.0	95.85* 71.43- 123.70	< 0.001
testosterone	ng/ml	0.26 0.17–0.43	0.15 0.11–0.27	0.28 0.24- 0.36	0.22 0.18- 0.33	0.21 0.13- 0.29	0.17 0.09- 0.27	
Free testosterone	pg/ml	0.20 0.13–0.83	0.22 0.09- 055	0.88 0.32- 0.9	0.27 0.17- 0.54	0.21 0.15- 0.50	0.12 0.08- 0.33	
DHEAS	µg/ml	119.5 80.5- 171.5	71 75.5- 163	180 100- 233.5	105.5 94- 199.5	113.5 73.8- 163	114 65.5- 148.5	
androstenedione	ng/ml	2.95 0.94- 2.00	0.6 0.58- 1.17	1.32 0.96- 1.52	1.09 0.69- 1.09	0.82 0.6- 1.05	0.79 0.43- 0.86	
androstenediol	ng/ml	0.54 0.33–0.71	0.25 0.22- 0.44	0.42 0.36- 0.55	0.33 0.35- 0.61	0.35 0.28- 0.56	0.55 0.25- 0.51	
estradiol	pg/ml	126.5 85.5-176.5	70 39- 115.5	64 45.5- 91.5	43.5 27.3- 85	12.5 5.0- 29	12.5 5.0- 34.5	
estrone	pg/ml	69.85 45.1-119.2	23.1 25.3- 74.5	58.1 32.4- 66.6	26.5 20.3- 49.9	16.6 13.5- 24	17 12.5- 21.3	

Group A: mid reproductive stage, Group B: late reproductive stage, Group C: early menopausal transition, Group D: late menopausal transition, Group E: very early post menopause, Group F: early postmenopause. Age, height, weight, BMI, LH and FSH are shown as medians. LH/FSH ratio is shown as a mean. LH: luteinizing hormone, FSH: follicle-stimulating hormone

### Levels of LH and FSH in premenopausal women, women during the menopausal transition and postmenopausal women

Levels of LH and FSH in premenopausal women, women during the menopausal transition and postmenopausal women are shown in Fig. 1. Both LH and FSH levels were significantly high in Groups D, E and F compared to those in Group A. In addition, LH level tended to be high in Group C compared to that in Group A (p = 0.062).

FSH level continued to high after Group D, but LH level reached a plateau at Group E (Fig. 1A and B). There was no significant difference in the LH/FSH ratio among the 6 groups (Table 1).

The box indicates values from the 25th percentile to the 75th percentile. The vertical line in the box indicates the median and the cross mark indicates the mean. Vertical lines represent minimum and maximum values. * p < 0.05 vs. Group A. # p = 0.06 vs. Group A.

Group A: mid reproductive stage, Group B: late reproductive stage, Group C: early menopausal transition, Group D: late menopausal transition, Group E: very early post menopause, Group F: early postmenopause.

### Correlations of LH with reproductive hormones

**Fig. 2:**
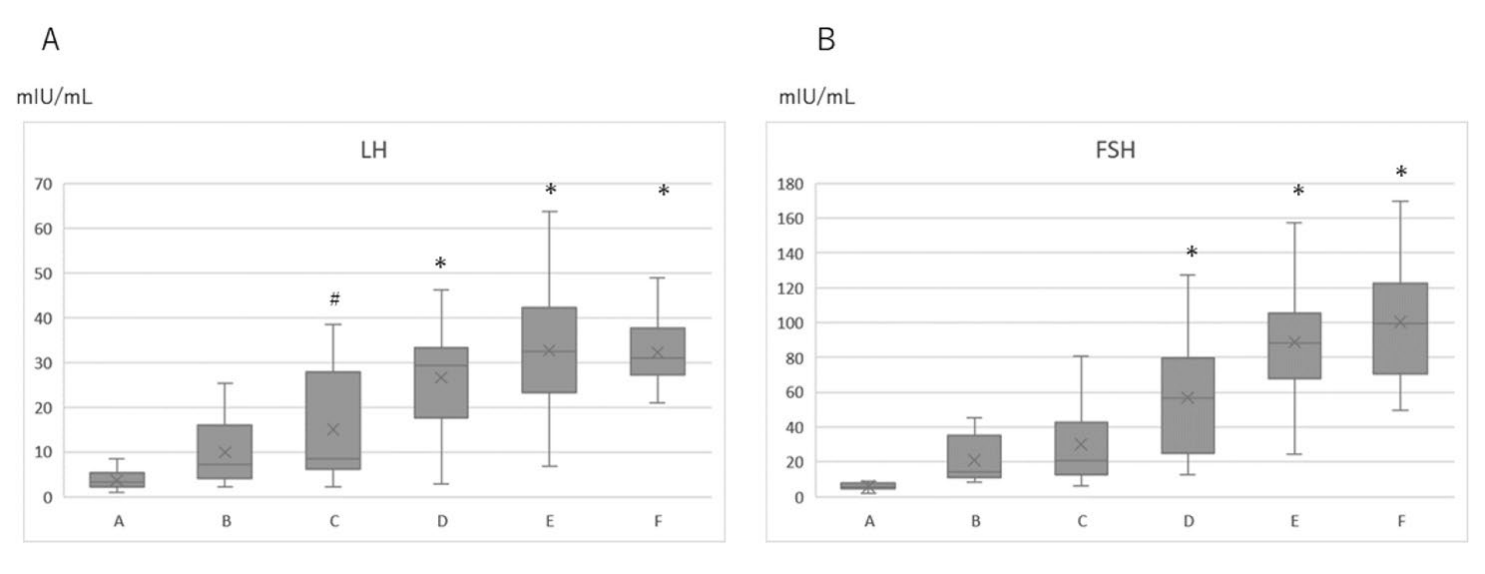
LH and FSH levels in premenopausal women, women during the menopausal transition and postmenopausal women

As can be seen in Table 2, in Group A, LH level showed significant and positive correlations with levels of androstenedione and estrone (r = 0.747, p = 0.003; r = 0.782, p = 0.038). In Group D, LH level was positively associated with levels of T and free T (r = 0.356, p = 0.036 and r = 0.414, p = 0.041, respectively) and was negatively associated with estradiol level (r=-0.484, p = 0.003). Positive associations between LH and FSH were found in Groups B, C, D and F (r = 0.713, p < 0.001; r = 0.776, p < 0.001; r = 0.692, p < 0.001; r = 0.688, p < 0.001), and a tendency for a positive correlation between LH and FSH was found in Group E (r = 0.358, p = 0.052) (Table 2). Significant correlations are also shown as scatterplot figures (Fig. 2A).

**Table 2:** Correlations of LH with reproductive hormones

	Group A	Group B	Group C	Group D	Group E	Group F
testosterone	R = 0.223 P = 0.331	R=-0.033 P = 0.883	R=-0.037 P = 0.867	**R = 0.356** **P = 0.036**	R=-0.004 P = 0.985	R=-0.004 P = 0.978
Free testosterone	R = 0.233 P = 0.309	R = 0.216 P = 0.335	R=-0.180 P = 0.410	**R = 0.414** **P = 0.041**	R=-0.205 P = 0.277	R=-0.153 P = 0.332
DHEAS	R = 0.235 P = 0.304	R=-0.051 P = 0.827	R = 0.234 P = 0.283	R = 0.295 P = 0.090	R = 0.136 P = 0.480	R = 0.076 P = 0.632
androstenedione	**R = 0.747** **P = 0.003**	R = 0.000 P = 1.000	R = 0.473 P = 0.142	R=-0.010 P = 0.970	R = 0.024 P = 0.955	R = 0.000 P = 1.000
androstenediol	R=-0.146 P = 0.688	R=-0.071 P = 0.867	R = 0.052 P = 0.813	R = 0.250 P = 0.516	R=-0.204 P = 0.279	R=-0.018 P = 0.907
estradiol	R = 0.427 P = 0.053	R = 0.166 P = 0.459	R = 0.300 P = 0.433	**R=-0.484** **P = 0.003**	R=-0.214 P = 0.645	R = 0.533 P = 0.139
estrone	**R = 0.782** **P = 0.038**	R = 0.048 P = 0.911	R=-0.217 P = 0.576	R=-0.095 P = 0.823	R = 0.501 P = 0.116	R=-0.009 P = 0.979
FSH	R = 0.233 P = 0.309	**R = 0.713** **P = 0.000**	**R = 0.776** **P = 0.000**	**R = 0.692** **P = 0.000**	R = 0.358 P = 0.052	**R = 0.688** **P = 0.000**

Spearman correlation coefficients were used to evaluate the correlations of LH with reproductive hormones for each group

Group A: mid reproductive stage, Group B: late reproductive stage, Group C: early menopausal transition, Group D: late menopausal transition, Group E: very early post menopause, Group F: early postmenopause

DHEAS: dehydroepiandrosterone sulfate, T: testosterone FSH: follicle-stimulating hormone

### Correlations of FSH with reproductive hormones

In Groups C and D, FSH level showed a significant and negative association with estradiol level (r=-0.432, p = 0.039 and r=-0.720, p < 0.001, respectively) but not with estrone level. FSH level was not associated with testosterone, DHEAS, androstenediol or androstenedione (Table 3). Significant correlations are also shown as scatterplot figures (Fig. 2B).

**Table 3:** Correlations of FSH with reproductive hormones

Stage	Group A	Group B	Group C	Group D	Group E	Group F
testosterone	R=-0.085 P = 0.715	R=-0.163 P = 0.469	R=-0.059 P = 0.789	R = 0.145 P = 0.407	R=-0.159 P = 0.402	R=-0.030 P = 0.853
Free testosterone	R=-0.021 P = 0.929	R = 0.120 P = 0.595	R=-0.079 P = 0.719	R = 0.135 P = 0.438	R=-0.334 P = 0.071	R=-0.180 P = 0.253
DHEAS	R = 0.067 P = 0.772	R=-0.065 P = 0.780	R = 0.354 P = 0.098	R = 0.302 P = 0.082	R=-0.159 P = 0.411	R = 0.076 P = 0.632
androstenedione	R=-0.307 P = 0.308	R = 0.013 P = 0.965	R = 0.227 P = 0.502	R=-0.216 P = 0.405	R = 0.048 P = 0.911	R=-0.393 P = 0.383
androstenediol	R = 0.129 P = 0.723	R=-0.024 P = 0.955	R=-0.275 P = 0.509	R=-0.233 P = 0.546	R=-0.500 P = 0.253	R = 0.000 P = 1.000
estradiol	R=-0.318 P = 0.160	R=-0.222 P = 0.321	**R=-0.432** **P = 0.039**	**R=-0.720** **P = 0.000**	R=-0.158 P = 0.406	R = 0.015 P = 0.925
estrone	R=-0.309 P = 0.500	R=-0.167 P = 0.693	R=-0.600 P = 0.088	R=-0.452 P = 0.260	R = 0.209 P = 0.537	R = 0.300 P = 0.370
LH	R = 0.233 P = 0.309	**R = 0.713** **P=0.000**	**R = 0.776** **P = 0.000**	**R = 0.692** **P = 0.000**	R = 0.358 P = 0.052	**R = 0.688** **P = 0.000**

Spearman correlation coefficients were used to evaluate the correlations of FSH with reproductive hormones and cortisol for each group

Group A: mid reproductive stage, Group B: late reproductive stage, Group C: early menopausal transition, Group D: late menopausal transition, Group E: very early post menopause, Group F: early postmenopause

DHEAS: dehydroepiandrosterone sulfate, T: testosterone, LH: luteinizing hormone

Significant correlations between LH and reproductive hormones are shown in Fig. 2A. In Group D, LH level was positively associated with levels of T and free T and was negatively associated with estradiol level. Significant correlations between FSH and reproductive hormones are shown in Fig. 2B. In Groups C and D, FSH level showed a significant and negative association with estradiol level.

## Discussion

In the present study, we found that the associations of LH and FSH with reproductive hormones differ according to stages of the menopausal transition.

Women in Group A had a normal range of FSH levels. However, the ages of women in Group A ranged from 41.6 to 46.5 years and gradually approached the age for menopause. The fact that the delta-4 pathway for synthesis of androstenedione and estrone acts well along with actions of 17β-HSD and 3β-HSD in women in that age range is thought to be the reason for the significant correlations of LH level with estrone and androstenedione levels in Group A. In women in Group A, the delta-4 pathway in the ovary may have been maintained due to a balance between LH and FSH.

In Group B, in which ovarian function had begun to decrease, there was no significant association of LH level with androstenedione or estrone level. Through an increase in FSH level, a relationship in which the balance between LH and FSH was maintained in Group A was considered to attenuate, and the shift might be beginning toward the delta-5 pathway. In addition, stimulation of DHEAS production in the ovary by augmentation of CYP17A1 activity due to an increase in LH may be the reason for the disappearance of correlations of LH with androstenedione and estrone. A correlation between LH and FSH was found with increasing FSH level.

In several studies, fibrosis in the stroma was observed in the ovary and associations between LH and androgens were found in women approaching menopause. Reproductive age-associated fibrosis was found in the stroma of ovaries in mice and humans [22, 23]. Matt et al. reported that alterations in hypothalamic-pituitary function such as a prolonged interpulse interval of LH and increased LH pulse width were found in middle-aged women with a mean age of 42.6 years [24]. A tripartile relationship among increase in LH, increase in androgens and fibrosis in the stroma in the ovary was also found in women with polycystic ovary syndrome (PCOS) [25]. Wickenheisser et al. reported that CYP17 gene expression increased for biosynthesis of androgens in theca interna cells in women with PCOS [17]. Moran et al. reported that women with PCOS who had an excess of adrenal androgen had significantly higher activity of CYP17 than that in women with PCOS who did not have an excess of adrenal androgen [16]. Thus, stimulation of DHEAS production by activation of CYP17A1 induces a hyperandrogenic state in women with PCOS [16, 17]. The results of those previous studies suggest that there are changes such as fibrosis in the ovarian stroma and transient increases in LH and androgens with aging. In Group C, production of androgen may have been started by the effect of increased LH level and a transition from the delta-4 pathway to the delta-5 pathway may have been proceeding. Negative correlations between FSH and estradiol were found to be significant in Group C and remarkable in Group D. When women enter into the periods of Group C and Group D, which indicate menopausal transition, the negative correlation between estradiol and FSH might become stronger due to the remarkable decline in estradiol level. As shown in Table 3, LH and FSH were negatively correlated with estradiol in Group D. In other words, estradiol levels decreased while levels LH and FSH increased in Group D, and we speculate that the site for production of steroid hormones may move from the ovary to the adrenal gland. Around the stage in Group D, changes in bone metabolism and lipid metabolism might be mainly involved in the decrease in estradiol level.

Murayama et al. reported that theca cells pretreated with a high concentration of LH showed increased CYP17 gene expression [18]. Oktem et al. reported that FSH up-regulated the mRNA expression of 17β-HSD and 3β-HSD in granulosa cells [19]. It has been reported that production of DHEAS is stimulated by an increase in LH in the adrenal gland in postmenopausal women [26]. In addition, production of DHEAS has been reported to be involved in CYP17A1 activity in the normal adrenal gland [27]. Due to an increase in LH in the late menopausal transition, we considered that CYP17A1 activity increases via LH receptors in the adrenal gland and the conversion from pregnenolone to DHEAS is stimulated. DHEAS level tended to increase, but the difference was not statistically significant (p = 0.062), in the present study. However, in a previous study, a transient increase in DHEAS was found in the late menopausal transition [28]. In the present study, LH was significantly associated with total T and free T in Group D (late menopausal transition). As well as DHEAS production in response to LH stimulation, conversion to T by stimulation of 17β-HSD activity via FSH increase is considered to be involved in this significant correlation [19]. The positive relationships between level of LH and levels of androgens including T, free T and DHEAS may indicate that the delta-5 pathway is the main pathway in Group D. In the late menopausal transition, LH and FSH may act cooperatively on enzyme activities and stimulate reproductive hormone production.

**Fig. 3:**
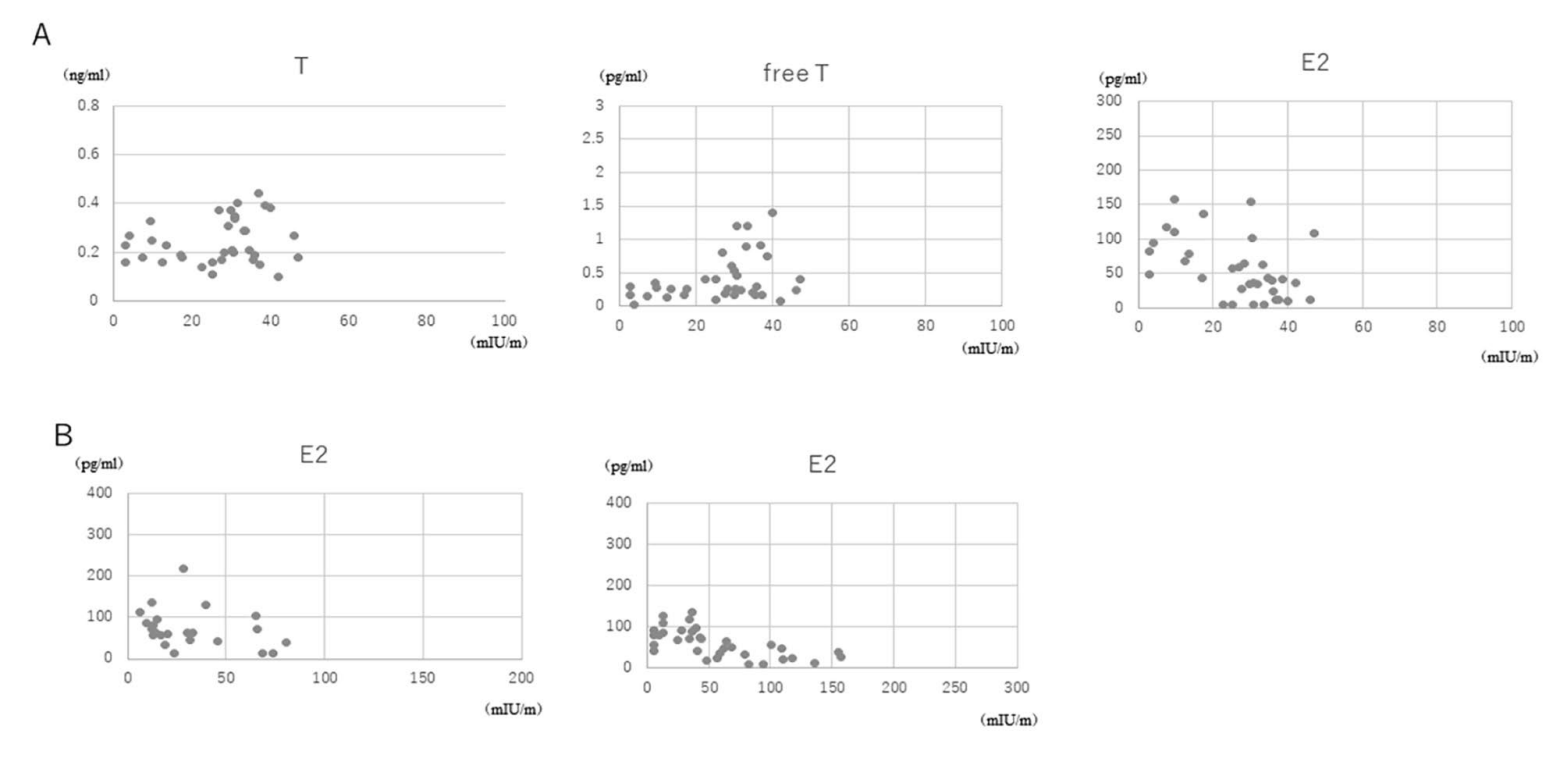
Scatterplots of correlations of LH or FSH with reproductive hormones

In Groups B, C, D and F, there were positive and significant associations between LH and FSH, but there was only a tendency for a correlation between LH and FSH in Group E. In the present study, in Group F, FSH level continued to increase, although LH level showed a plateau (Fig. 3). In groups B, C and D, both levels of LH and FSH increased in the same way. However, in group E, LH reached a plateau, but FSH continued to increase. Therefore, the time difference in hormonal levels in which a plateau was reached might be involved in the weak correlation between LH and FSH in Group E. The time difference in hormonal changes in which FSH reached a plateau later than LH might be involved in the weak correlation between LH and FSH in Group E. In addition, in Group E, the correlations of LH with T and free T shown in Group D disappeared. The reason may be the conversion from T to estradiol by an increase in aromatase through continuation of the increase in FSH, and this phenomenon was maintained in Group F.

In postmenopausal women, associations of FSH levels with the development of various diseases and with metabolism have been reported [29–31]. A high FSH level was shown to be associated with prevalence of vasomotor symptoms [32] and an increase in low-density lipoprotein cholesterol [5], and a low FSH level was shown to be associated with non-alcoholic fatty liver disease [33] and diabetes mellitus [29]. Serum FSH levels have been shown to be correlated with the rate of bone loss in perimenopausal women aged 42–52 years [34] and in postmenopausal women with a mean age of 81 years [35]. However, the associations that were investigated in those studies were for FSH levels, not LH levels, in postmenopausal women. The associations of FSH levels with lipid metabolism, carbohydrate metabolism and vascular function in postmenopause may be affected by the increase in androgen levels due to an increase in LH during the menopausal transition. Combined studies on LH levels and FSH levels from menopausal transition to postmenopause may be valuable.

Fibrosis in the stroma in the ovary and increases in LH and androstenedione occur in women with aging. Stimulation of 17β-HSD activity due to an increase in FSH might be regulated so as to prevent an excessive increase in androgen levels, particularly in postmenopausal women with high FSH levels. We did not examine the associations of LH and FSH with lipid metabolism and insulin resistance in each stage of the menopausal transition. Studies on associations of LH with the development of various diseases and with metabolism should be carried out not only for women in postmenopause but also for women during the menopausal transition.

There are some limitations in this study. The sample size in the present study might be insufficient for a generalization for all Japanese women. Further study with a large sample size is needed. This study was a cross-sectional study. Thus, a causal relationship needs to be clarified in a longitudinal study. In addition, measurements of various enzymes related to the production of hormones may be important to clarify individual differences in enzyme activities. In the present study, total circulating reproductive hormones were measured, and we could not separate ovary-derived reproductive hormones and adrenal gland-derived reproductive hormones.

## Conclusion

The associations of LH and FSH with reproductive hormones are different depending on the stage of the menopausal transition.

## Data Availability

All data and materials are available upon reasonable request from the corresponding author.
